# Effect of Internal Pressure on the Layered Microstructural Evolution of N36 Zirconium Alloy Cladding Tubes During LOCA Biaxial Creep at 900 °C

**DOI:** 10.3390/ma19153348

**Published:** 2026-08-06

**Authors:** Zhien Ning, Xu Ji, Wei Zhang, Jijun Yang, Linjiang Chai

**Affiliations:** 1 Key Laboratory of Radiation Physics and Technology of Ministry of Education, Institute of Nuclear Science and Technology, Sichuan University, Chengdu 610064, China; 2 The First Sub-Institute, Nuclear Power Institute of China, Chengdu 610041, China; 3 College of Materials Science and Engineering, Chongqing University of Technology, Chongqing 400054, China

**Keywords:** zirconium alloy cladding tube, LOCA, biaxial creep, internal pressure, microstructural evolution

## Abstract

The effect of internal pressure on the layered microstructural evolution of N36 zirconium alloy cladding tubes was systematically studied under simulated loss-of-coolant accident (LOCA) biaxial creep conditions at 900 °C. The tested specimens were characterized by electron channeling contrast imaging, energy-dispersive X-ray spectroscopy, electron backscatter diffraction, and transmission electron microscopy. The results show that all specimens formed a typical layered cross-sectional structure consisting of an oxide film, an oxygen-rich α-Zr (α(O)) layer, and a prior-β transformed layer. The thickness of the α(O) layer and the oxygen diffusion depth changed markedly with internal pressure. The thickness of the α(O) layer was approximately 21 μm for the 0.8 MPa specimen and 11 μm for the 1.9 MPa specimen, respectively. The lower-pressure specimen exhibited a wider oxygen-affected region, whereas the higher-pressure specimen showed a steeper oxygen gradient. In the prior-β transformed layer, lath-like α structures formed under both conditions, but their spatial arrangement and orientation distribution were different. Under lower internal pressure, the laths were more regularly arranged and showed a more complete colony structure. Under higher internal pressure, the laths were more interwoven, and the orientation distribution became more scattered. Meanwhile, the high-pressure specimen retained a higher local orientation gradient and a higher degree of lattice distortion. These results indicate that the above microstructural differences mainly arise from the effect of internal pressure on the high-temperature exposure history. A higher internal pressure causes earlier instability of the specimen, thereby shortening the effective time for oxygen diffusion and microstructural evolution, rather than directly changing the oxidation or phase transformation process.

## 1. Introduction

Zirconium alloys are widely used as fuel cladding materials in light water reactors because of their low thermal neutron absorption cross-section, good corrosion resistance, and irradiation stability [1,2,3]. Under LOCA conditions, the cladding experiences rapid heating, high-temperature steam oxidation, and ballooning and instability under internal pressure. Its structural integrity is therefore controlled not only by normal corrosion behavior but also by the coupled effects of high-temperature oxidation, creep deformation, and phase transformation [4,5,6,7]. Therefore, revealing the microstructural evolution of zirconium alloys under LOCA conditions, especially the formation and control of layered structures, is important for understanding cladding failure.

Previous studies have shown that after oxidation in high-temperature steam and subsequent cooling, zirconium alloys usually form a layered structure along the wall thickness. From the outside to the inside, this structure consists of an oxide film, an α(O) layer, and a prior-β transformed layer [8,9,10]. This layered structure comes from oxygen diffusion toward the metal side and the resulting gradient in phase stability. The near-surface region is stabilized as the α phase because of its high oxygen content, while the inner region with lower oxygen content is in the β phase field at high temperature and then undergoes β → α transformation during cooling [11,12]. This composition-gradient-driven microstructural partitioning not only determines the different mechanical responses along the wall thickness but also provides an important microstructural basis for evaluating cladding performance after LOCA.

Among LOCA-related temperature ranges, around 900 °C is a sensitive window for microstructural evolution. On the one hand, this temperature is high enough to promote oxygen diffusion into the matrix and form a clear α(O) layer. On the other hand, the β-phase stability of the inner low-oxygen region and the β → α transformation during cooling also become important [9,13,14,15]. Therefore, the microstructures obtained at 900 °C usually contain information related to both diffusion control and phase transformation control, providing suitable conditions for analyzing their coupling effect.

In addition to temperature, the cladding is also subjected to a biaxial stress state during LOCA, which is produced by internal pressure and axial constraint [6]. Previous studies have mainly focused on the effect of internal pressure on ballooning deformation and burst behavior [16,17]. From a microstructural point of view, however, the stress state may also affect the transformed structure by changing interface migration, lath morphology, and local orientation distribution [18,19]. During β → α transformation in zirconium alloys, the Burgers orientation relationship is usually followed. The formation of different α variants and their orientation combinations are closely related to transformation kinetics and the local stress state [20,21,22,23,24]. Therefore, the morphology and orientation features of lath structures in the prior-β transformed layer reflect not only the temperature–time history, but may also be affected by the stress condition.

Although many studies have been carried out on oxidation behavior, ballooning and rupture, and phase transformation of zirconium alloys under LOCA conditions [25], some issues remain unclear. First, systematic microstructural characterization of the layered structure along the thickness direction is still limited for the key temperature region around 900 °C. Second, stress effects have mostly been discussed from the perspective of macroscopic mechanical behavior. Direct microstructural evidence is still lacking on how stress affects oxygen diffusion depth, lath regularity, and the retention of orientation gradients by changing the high-temperature exposure history and local stress state.

Based on these considerations, N36 zirconium alloy cladding tubes were used in this study. Simulated LOCA biaxial creep tests were carried out in steam at 900 °C under different internal pressures. The layered cross-sectional microstructures after testing were systematically characterized by electron channeling contrast imaging (ECCI), energy-dispersive X-ray spectroscopy (EDS), electron backscatter diffraction (EBSD), and transmission electron microscopy (TEM). The main focus was placed on the thickness and morphology of the α(O) layer, as well as the lath structure and orientation distribution in the prior-β transformed layer under different internal pressures. The role of internal pressure in controlling the formation and evolution of the layered structure through its effect on the high-temperature exposure history was further discussed.

## 2. Experimental

The as-received material was recrystallization-annealed N36 cladding tube with an outer diameter of 9.5 mm, an inner diameter of 8.36 mm, and a wall thickness of 0.57 mm. The chemical composition of the N36 cladding tube is listed in Table 1. The initial microstructure of N36 cladding tubes in the recrystallization-annealed state generally consists of equiaxed α-Zr grains with a high recrystallization fraction [26]. For the high-temperature biaxial creep tests under simulated LOCA conditions, the cladding tube was cut into 220 mm-long tube sections. ZnO pellets were then filled into the tubes, and both ends were sealed.

**Figure 1:**
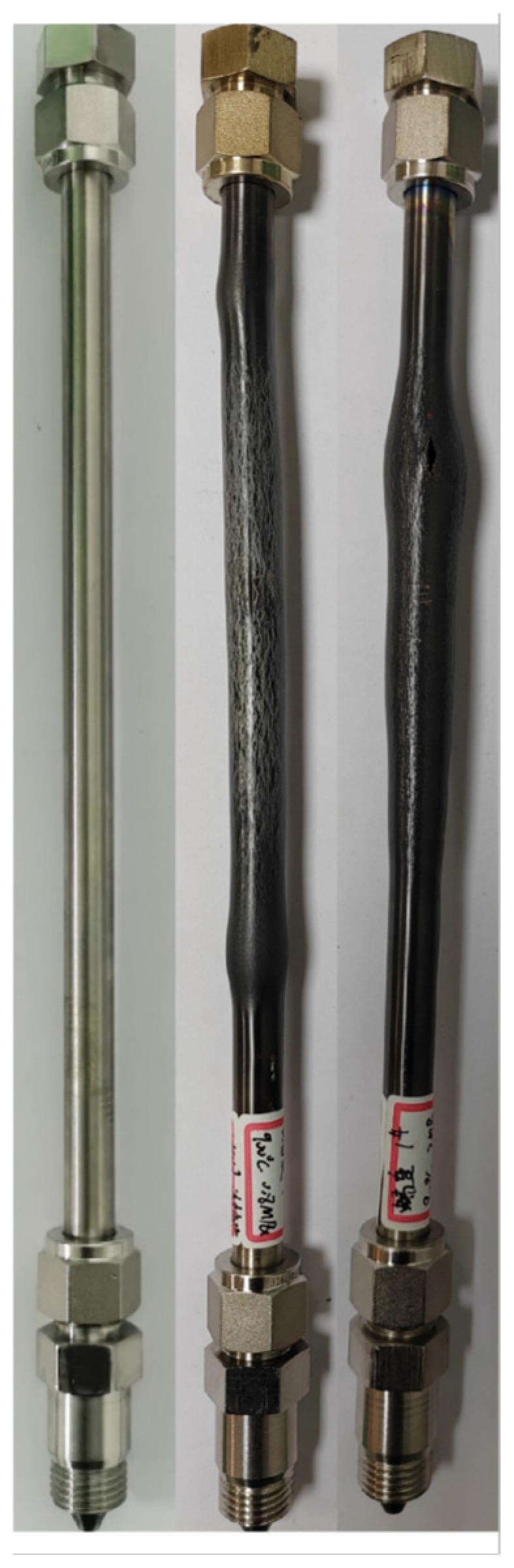
Images of the experimental specimens (from left to right: the as-received, 900 °C–0.8 MPa, and 900 °C–1.9 MPa specimens).

**Figure 2:**
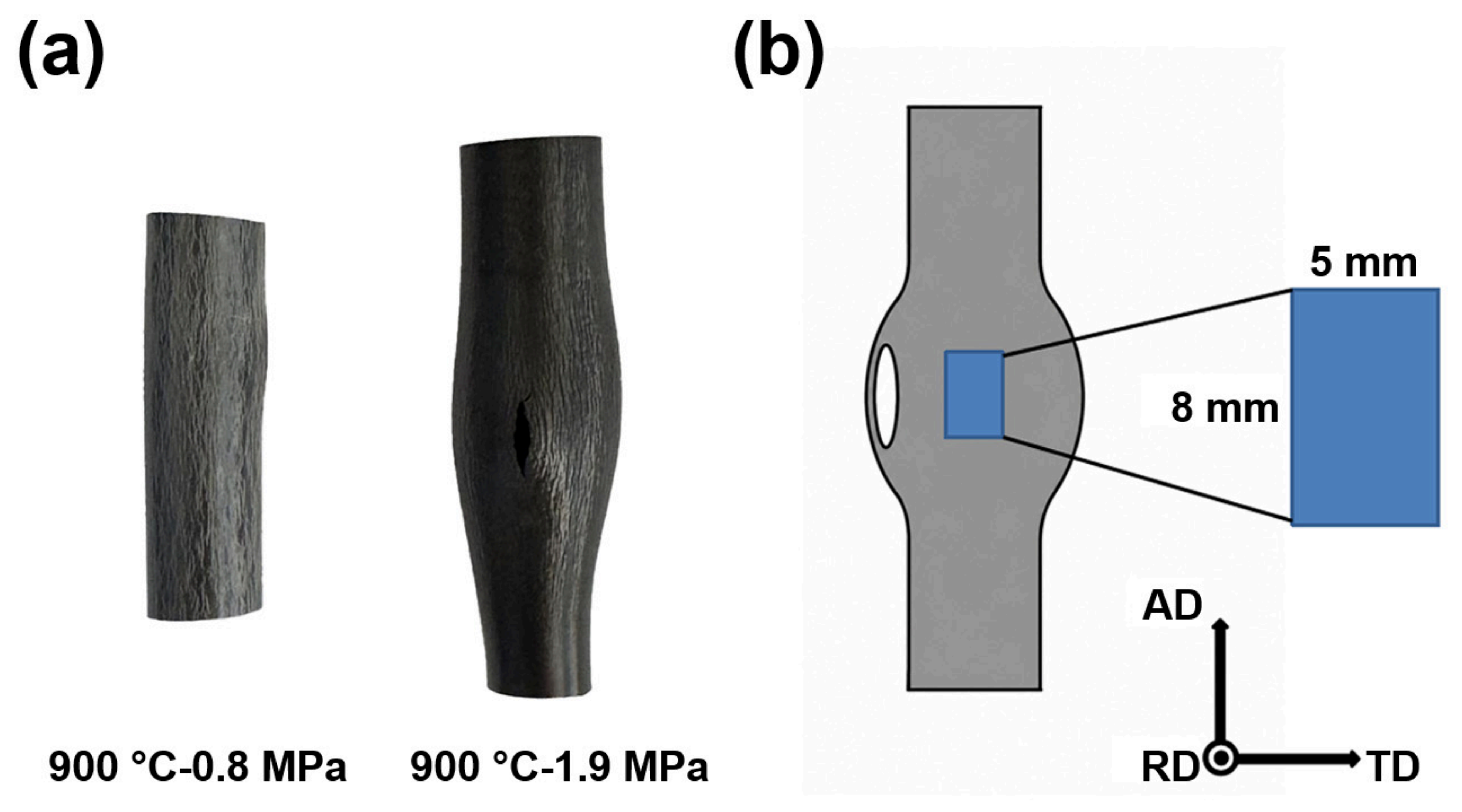
(**a**) Macroscopic morphologies of the sectioned tubes after LOCA biaxial creep tests; (**b**) schematic illustration of the specimen locations cut from the sectioned tubes.

The tests were performed using a shielded LOCA biaxial creep testing facility developed by the Nuclear Power Institute of China. The system enables simultaneous control of temperature, steam atmosphere, axial loading, and internal pressure. During testing, the specimen temperature, axial load, steam environment, and internal pressure were independently controlled to simulate the coupled thermo-mechanical conditions during LOCAs. During the test, the specimen was first placed in the reaction furnace, and a constant axial tensile load of 100 N was applied. After a steam atmosphere was established and filled the furnace, the specimen was heated to 900 °C and held at this temperature. During the holding stage, internal pressure was applied to the tube, with target pressures of 0.8 MPa and 1.9 MPa. These two pressure levels were selected to represent different pressurization conditions during simulated LOCA biaxial creep tests and to evaluate their effects on ballooning instability and microstructural evolution. After the target pressure was reached, the pressure was kept constant until the specimen ruptured. The test was then stopped immediately, and the specimen cooled in the residual steam atmosphere inside the furnace. The cooling rate was faster than that of conventional static furnace cooling. During the tests, the temperature and internal pressure were continuously monitored to ensure the stability of the simulated LOCA conditions. The present study focuses on the effect of internal pressure on the post-test layered microstructural evolution rather than the creep deformation behavior or constitutive response of the zirconium alloy. The macroscopic morphologies of the specimens after LOCA biaxial creep tests were recorded to evaluate the deformation characteristics under different internal pressures. As shown in Figure 1 and Figure 2a, both specimens exhibited obvious circumferential deformation after testing, and the maximum ballooning regions were selected for subsequent microstructural characterization. Compared with the 0.8 MPa specimen, the 1.9 MPa specimen showed more severe local ballooning, indicating a stronger deformation response under higher internal pressure. After the test, strip specimens with a size of 5 mm × 8 mm were cut along the transverse direction (TD) and axial direction (AD) from the maximum ballooning region (Figure 2b). The radial direction (RD) thickness was the thickness after ballooning and was measured separately.

The microstructures were characterized by ECCI, EDS, EBSD, and TEM. ECCI was carried out using a Zeiss Sigma HD field-emission scanning electron microscope (Carl Zeiss Microscopy GmbH, Jena, Germany). EBSD data were collected using a Thermo Fisher Apreo 2S field-emission scanning electron microscope (Thermo Fisher Scientific, Brno, Czech Republic) equipped with an Oxford NordlysMax^2^ detector (Oxford Instruments, High Wycombe, UK), and the data were processed using AZtec 5.1 (version number 5.1.7829.1) and HKL Channel 5 (version number 5.11.20405.0) software. TEM foils were prepared by focused ion beam (FIB). A Pt protection layer was first deposited on the target area to reduce surface damage during ion-beam cutting and thinning. Trenches were then milled on both sides of the target region using a Ga^+^ ion beam to obtain a thin foil. The foil was lifted out by a micromanipulator and fixed onto a TEM grid. It was then thinned step-by-step by lowering the ion-beam accelerating voltage and current until an electron-transparent region suitable for TEM observation was obtained. TEM observation was performed using an FEI Talos F200X microscope (Thermo Fisher Scientific, Brno, Czech Republic) equipped with an EDS detector (Thermo Fisher Scientific, Brno, Czech Republic). Before ECCI and EBSD characterization, all cross-sectional specimens were mechanically ground using SiC papers and then electropolished at −30 °C in a solution of 10% HClO_4_ + 90% C_2_H_5_OH. The polishing parameters were 20 V and 45 s. In addition, because the oxide film on the specimen surface may detach during electropolishing, some ground specimens were directly polished by Ar ion milling using a Fischione 1061 system to better observe the cross-sectional features of the oxide film.

## 3. Results

**Figure 3:**
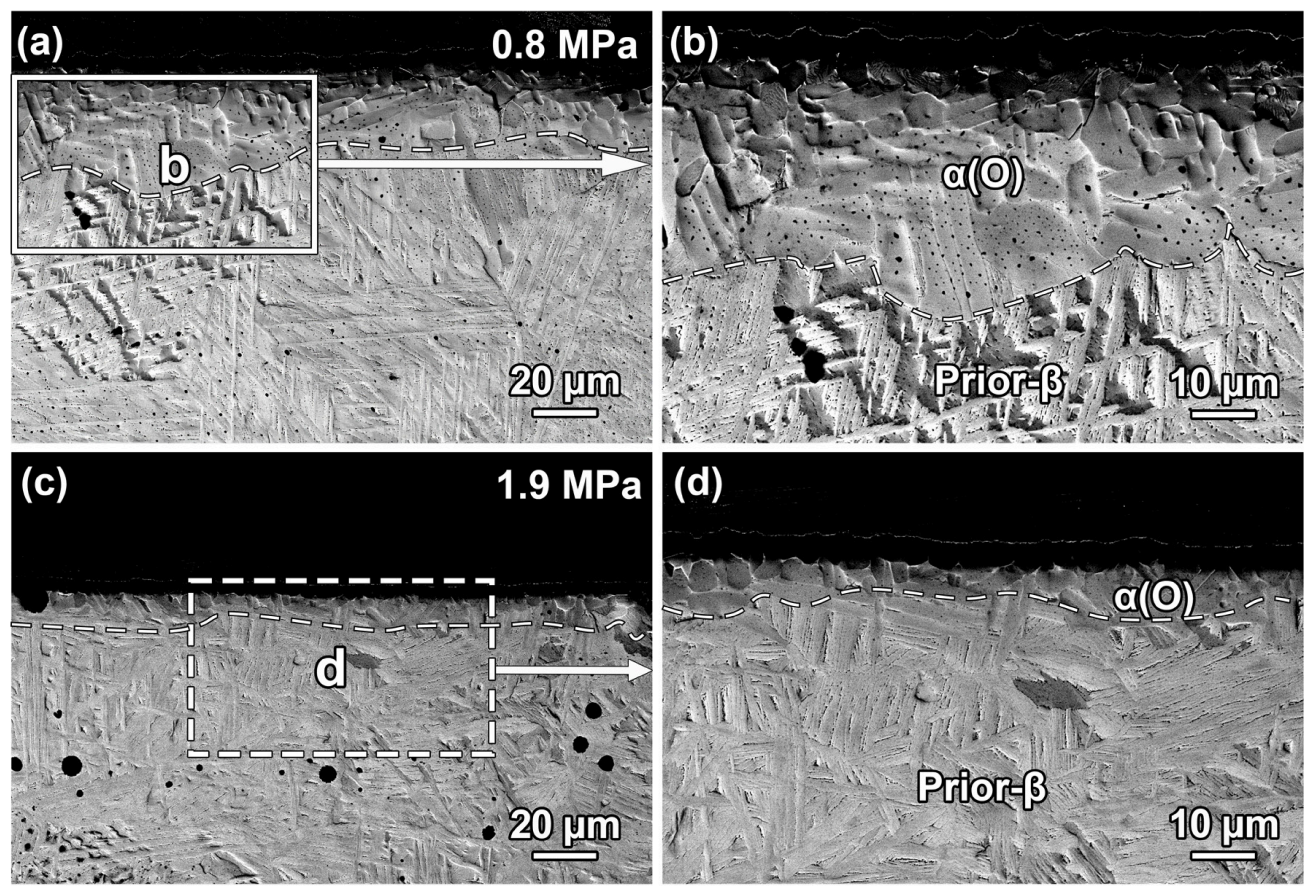
Cross-sectional (RD-AD) ECCI results of the (**a**,**b**) 0.8 MPa and (**c**,**d**) 1.9 MPa specimens after electropolishing.

Figure 3 shows the ECCI results of the cross-sections of the two specimens. Both specimens show a clear layered structure. The region above the dashed line is the α(O) layer, while the region below it is the prior-β transformed layer, namely the lath/lamellar region formed after cooling from the high-temperature low-oxygen region. Clear lamellar or lath-like contrast can be observed in the prior-β layer of both specimens, indicating that a marked transformed structure formed in the inner region at 900 °C. The morphology of the α(O)/prior-β interface differs clearly under different internal pressures. In the 0.8 MPa specimen, the α(O) layer is relatively thicker (average thickness of approximately 21 μm), and the layered interface is more uneven. By contrast, the α(O) layer in the 1.9 MPa specimen is thinner (average thickness of approximately 11 μm), and the interface is generally smoother. These results indicate that although a typical layered structure formed under both conditions, the change in internal pressure strongly affects the thickness of the α(O) layer and the interface morphology.

**Figure 4:**
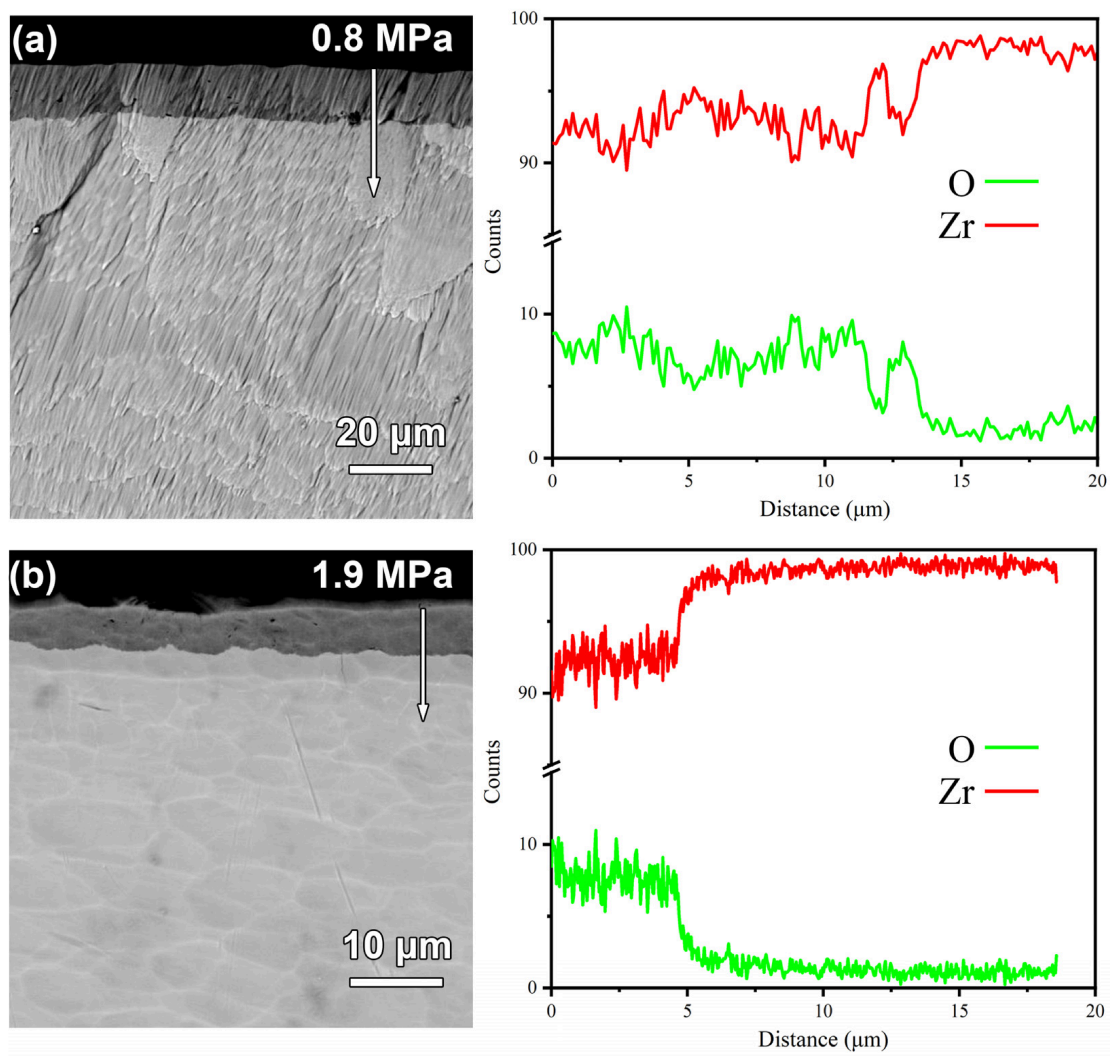
Cross-sectional (RD-AD) morphology and EDS results of the (**a**) 0.8 MPa and (**b**) 1.9 MPa specimens after Ar ion polishing.

It should be noted that the cross-sections shown in Figure 3 were prepared by electropolishing, which may cause the oxide film on the specimen surface to detach. To better observe the oxide film, the interfaces of specimens prepared directly by Ar ion polishing were further characterized, as shown in Figure 4. Compared with Figure 3, Figure 4 clearly shows the oxide film on the cross-sections of both specimens. From the outside to the inside, the structure consists of the oxide film, α(O) layer, and prior-β transformed region. The EDS line-scan results show that the O content reaches a high level in the outermost oxide film and then gradually decreases along the thickness direction. In contrast, the Zr content is reduced near the surface because of oxidation, and then gradually increases with depth until it becomes stable in the matrix. This elemental distribution indicates clear inward oxygen diffusion in both specimens, which agrees with the layered microstructure shown in Figure 3.

The elemental distribution along the thickness direction differs clearly under different internal pressures. In the 0.8 MPa specimen, the O content remains high near the surface and gradually decreases within about 10–15 μm. Correspondingly, the Zr content recovers to a stable level over a larger depth range. This suggests a wider oxygen diffusion-affected region and a gradual elemental gradient under this condition. By contrast, in the 1.9 MPa specimen, the O content decreases sharply at about 5 μm and almost returns to the matrix level within 7–8 μm. Meanwhile, the Zr content increases rapidly and then becomes stable. This indicates a smaller oxygen diffusion-affected depth in the matrix and a steeper elemental gradient. Together with the cross-sectional morphology in Figure 3, the surface affected region in the 1.9 MPa specimen is generally narrower, which is consistent with the rapid decrease in the O signal in the line-scan results. Overall, both specimens formed a typical “oxide film–α(O) layer–prior-β transformed region” structure. However, the 0.8 MPa specimen shows a wider oxygen diffusion-affected region and a more gradual elemental gradient, while the 1.9 MPa specimen shows a narrower oxygen diffusion-affected region and a steeper elemental gradient.

**Figure 5:**
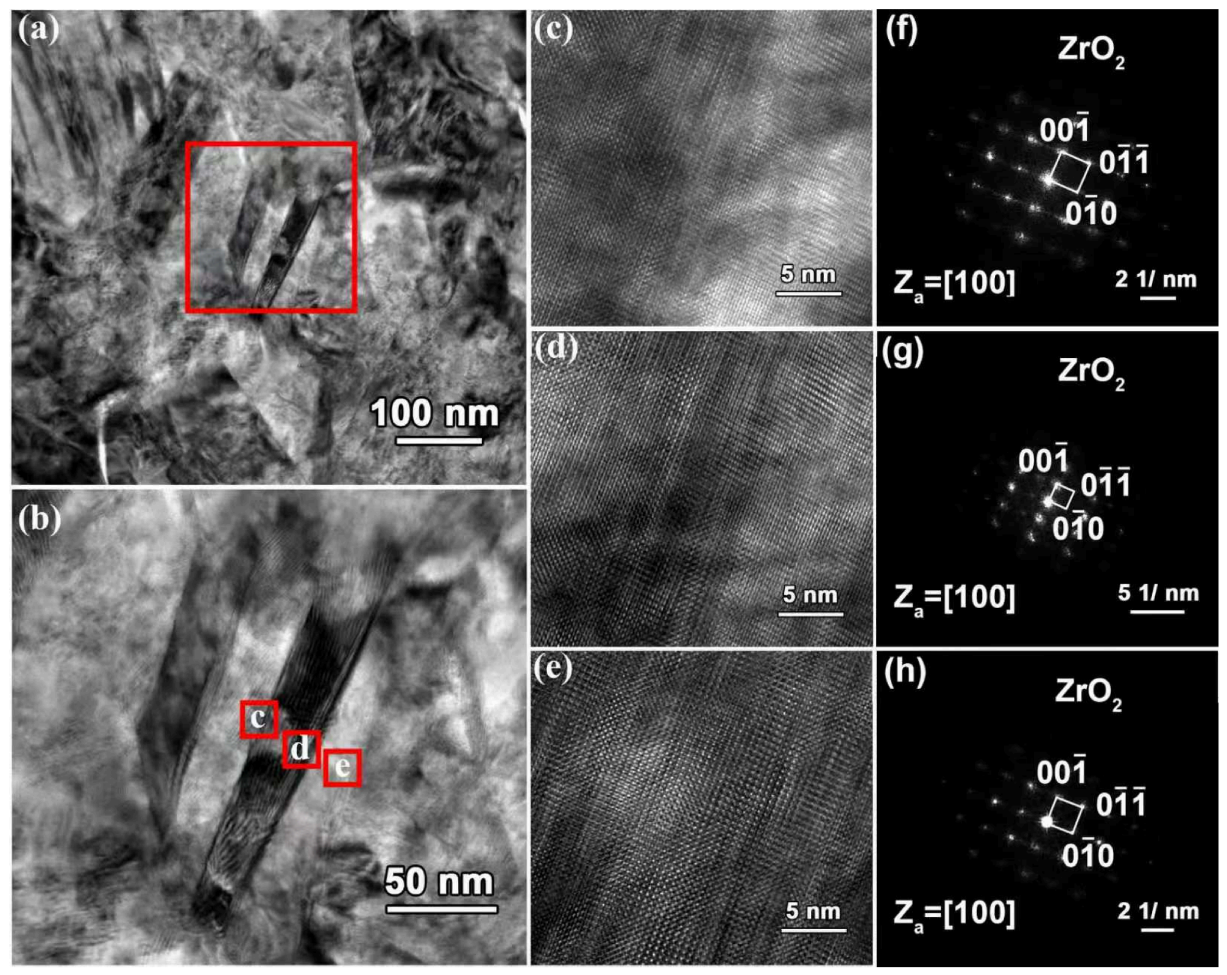
TEM characterization of the oxide film in the 0.8 MPa specimen: (**a**) typical grains in the oxide; (**b**) magnified image of the boxed region in (**a**); (**c**–**e**) HRTEM images of the boxed regions c–e in (**b**); (**f**–**h**) corresponding FFT patterns of (**c**–**e**).

The 0.8 MPa specimen was selected for TEM characterization of the typical oxide film structure near the surface, as shown in Figure 5. The low-magnification TEM image in Figure 5a shows that the oxide film is mainly composed of fine equiaxed grains, with some local variation in thickness. The enlarged image in Figure 5b further shows that the grains in the oxide film are small and display mixed equiaxed and columnar morphologies. The high-resolution images in Figure 5c–e and the corresponding FFT results show clear orientation changes across local interfaces. The FFT diffraction spots can be indexed as the (001), (010), (020), and (011) planes, indicating that the crystal structure in these regions is consistent with monoclinic ZrO_2_.

**Figure 6:**
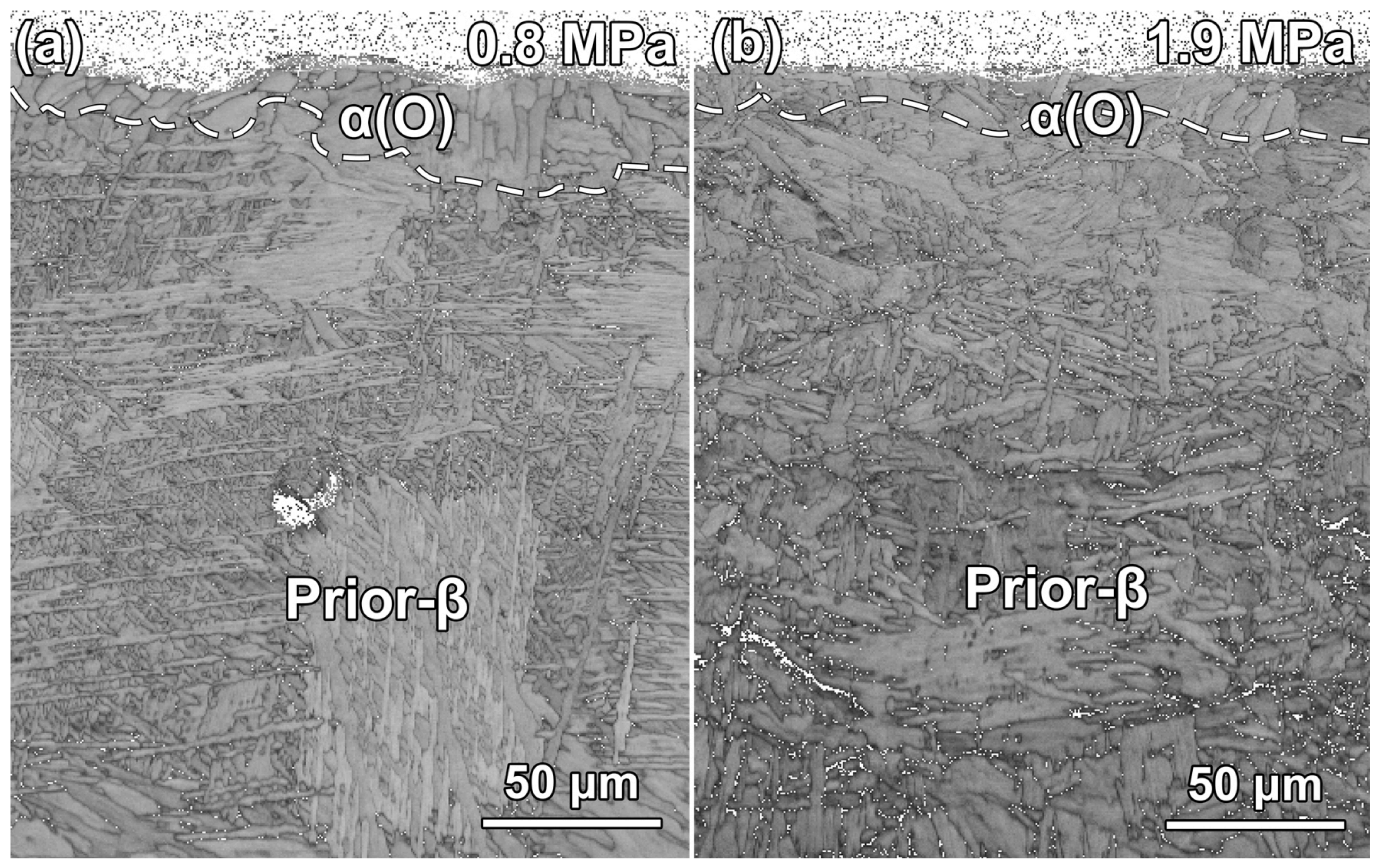
BC maps of the RD-AD cross-sections of different specimens: (**a**) 0.8 MPa and (**b**) 1.9 MPa.

Figure 6 shows the band contrast (BC) results of different specimens. Both cross-sections show a clear layered structure. The upper α(O) layer is mainly composed of equiaxed grains, while the lower prior-β layer mainly shows interlaced lath/lamellar structures, which are typical features of a transformed microstructure. The lath morphology in the prior-β layer changes with internal pressure. In the 0.8 MPa specimen, the laths are generally finer, and the outline of some prior-β grains can still be identified locally. This suggests that the transformed structure retains some geometric boundary information from the parent phase. By contrast, in the 1.9 MPa specimen, the lath bundles are clearer and some laths are relatively wider, while the prior-β grain outlines are less easy to identify.

**Figure 7:**
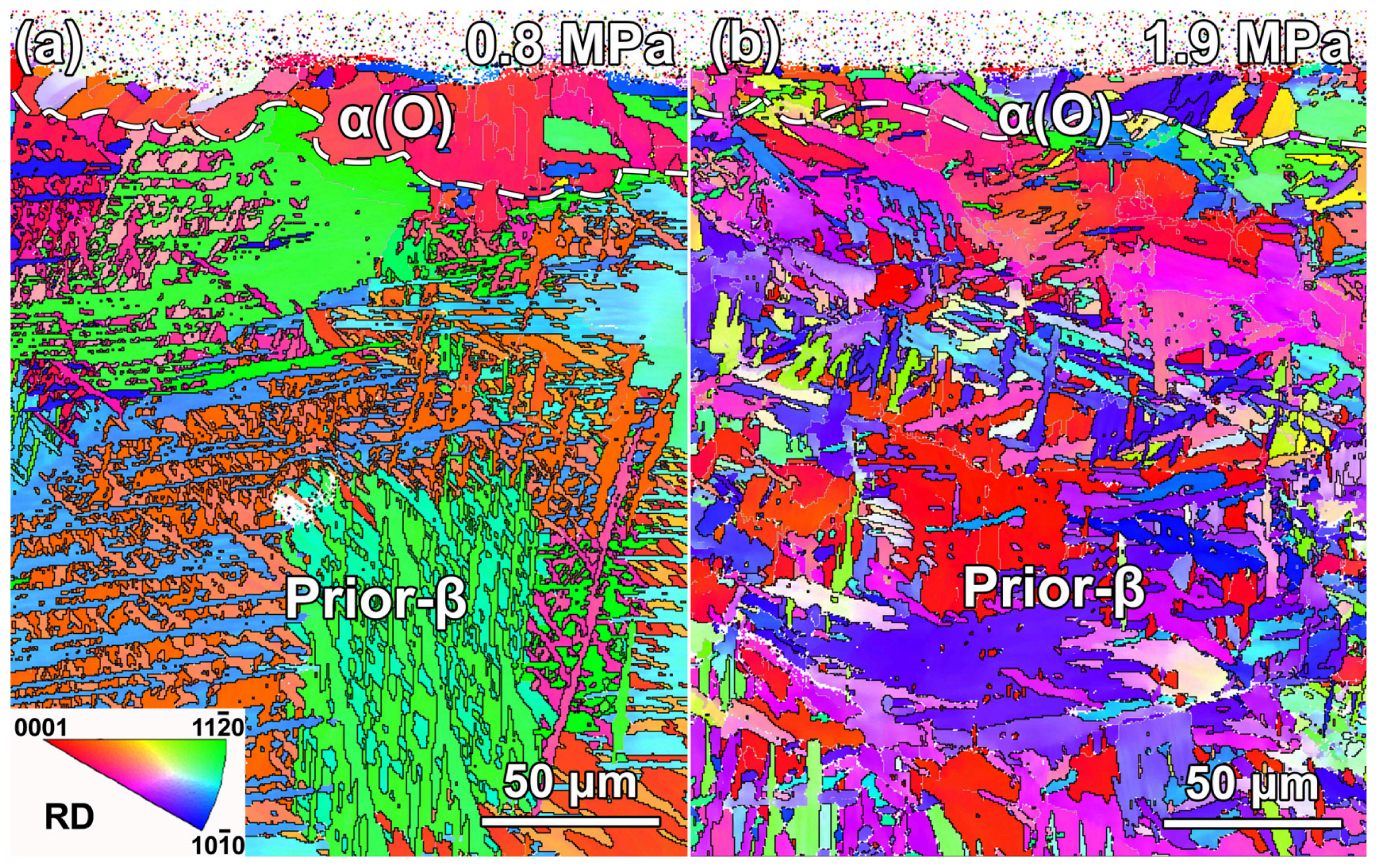
IPF maps of the RD-AD cross-sections of different specimens: (**a**) 0.8 MPa and (**b**) 1.9 MPa.

Figure 7 shows the inverse pole figure (IPF) maps corresponding to Figure 6. Low-angle boundaries (LABs, 2° < θ < 15°) and high-angle boundaries (HABs, θ > 15°) are shown as silver-gray and black lines, respectively. Both specimens show a clear layered structure. Below the unindexed oxide film is the α(O) layer. The dashed line marks the α(O)/prior-β interface, and the inner region is the prior-β transformed layer, which is mainly composed of lath-like α phase.

Under the 0.8 MPa condition (Figure 7a), the lath α phase shows a typical colony structure. Laths with similar colors are mostly arranged in nearly parallel bundles. Clear orientation changes and HABs are present between different lath colonies, showing distinct orientations. In addition, the prior-β grain-scale outlines can still be identified in some regions, indicating that the transformed structure partly retains the geometric boundary information of the parent phase. By contrast, under the 1.9 MPa condition (Figure 7b), the orientation connection among lath α regions is more complex. Although same-color lath bundles are still present, they are more often divided by laths with different orientations. Some local regions show stronger orientation fragmentation and more interwoven laths. At the same time, large single-color lath groups coexist with surrounding multi-orientation laths, indicating a more uneven distribution of lath/colony sizes and a more scattered orientation combination.

**Figure 8:**
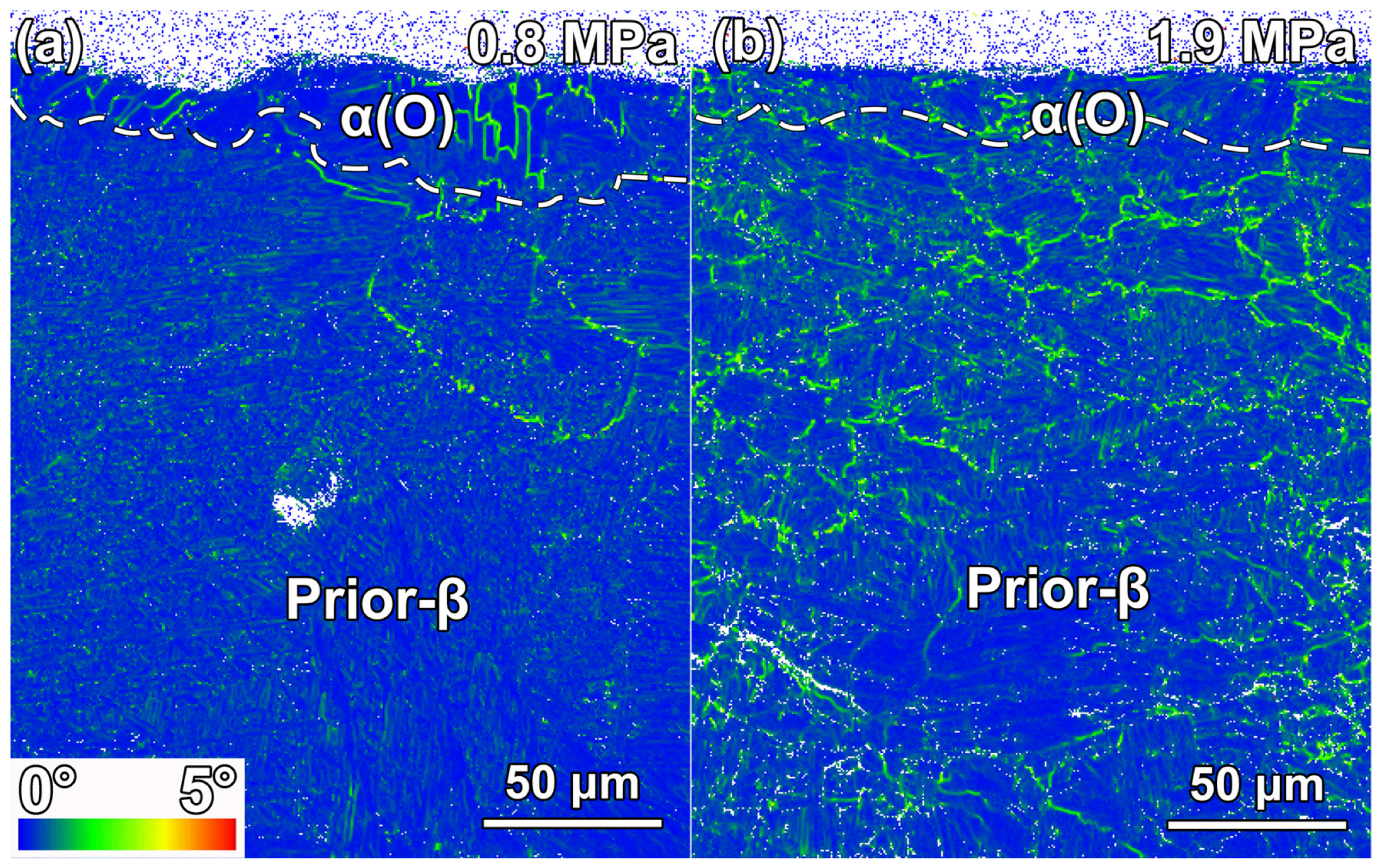
KAM maps of the RD-AD cross-sections of different specimens: (**a**) 0.8 MPa and (**b**) 1.9 MPa.

Figure 8 shows the KAM maps of the two specimens. Overall, both specimens are mainly composed of low-KAM regions, shown in blue. Medium- and high-KAM regions (shown in green) are mainly distributed near lath interfaces, grain boundaries, and lath intersections. This indicates that local orientation gradients are mainly concentrated near microstructural interfaces. Under the 0.8 MPa condition (Figure 8a), the average KAM value is 0.35. High-KAM regions are generally scattered and mainly limited to lath colony intersections and some local interfaces, suggesting a relatively low local orientation gradient. By contrast, under the 1.9 MPa condition (Figure 8b), the average KAM value increases to 0.48. High-KAM regions become more obvious and form a more continuous network along lath boundaries. Some band-like or blocky high-KAM regions can also be seen locally. This indicates that the 1.9 MPa specimen retains a higher local orientation gradient and has a more uneven distribution of internal distortion.

**Figure 9:**
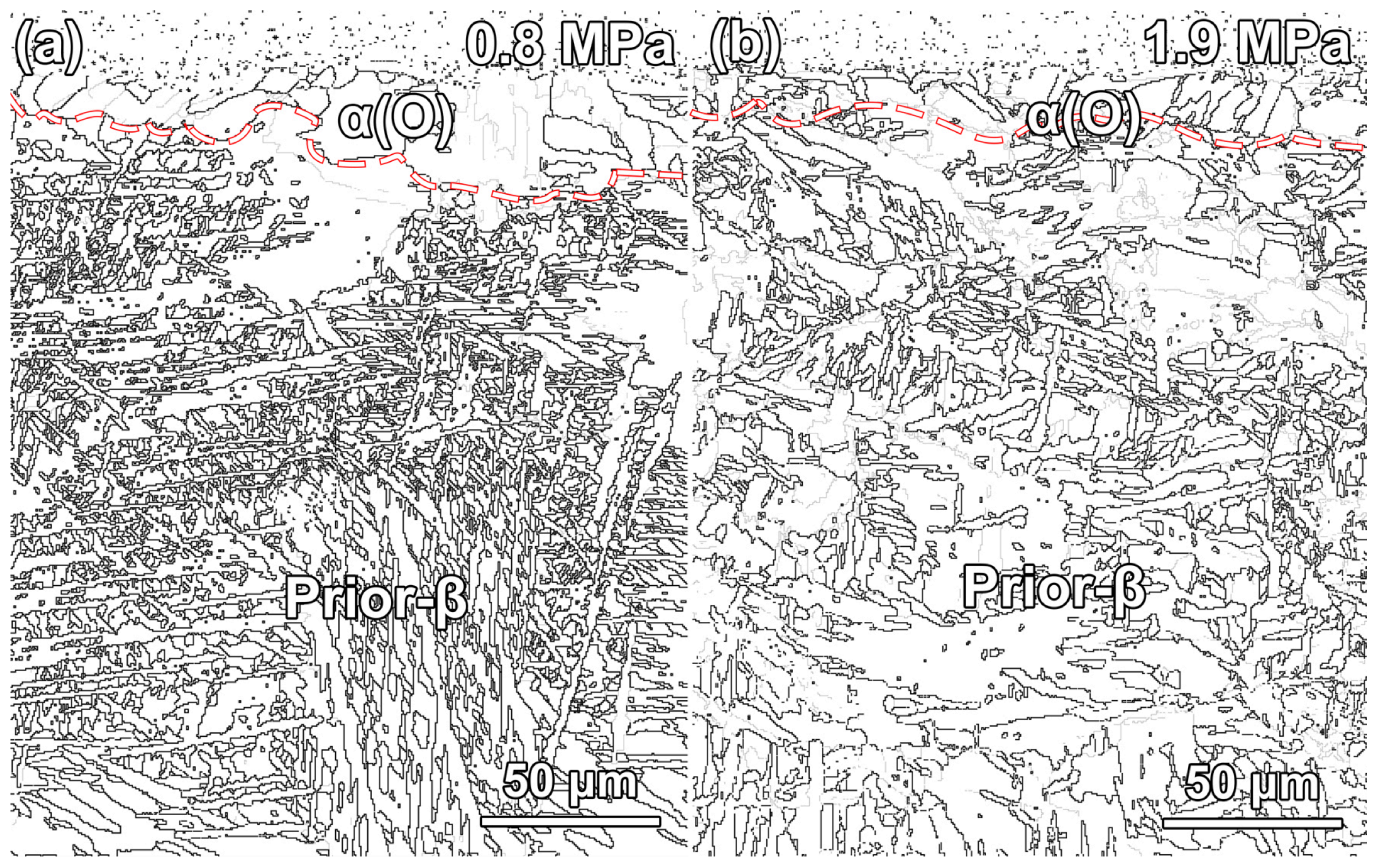
GB maps of the RD-AD cross-sections of different specimens: (**a**) 0.8 MPa and (**b**) 1.9 MPa.

As shown in Figure 9, the α(O) layer in both specimens mainly consists of a relatively uniform equiaxed grain-boundary network, while many lath interfaces with different orientations are present in the prior-β layer, forming typical packets and colonies. A comparison between the two internal pressures shows that the lath boundaries in the prior-β layer of the 0.8 MPa specimen are more densely distributed, and the orientation difference between lath colonies is clearer. In contrast, the 1.9 MPa specimen shows stronger lath colony interweaving and connection. In some local regions, laths with multiple orientations cut across each other, and the spatial distribution of the structure becomes more uneven.

The misorientation angle distributions further reveal the orientation differences between different layers. In the α(O) layer (Figure 9a), in addition to the main peak near 60°, obvious low-angle misorientations around 5–10° and high-angle misorientations close to 90° can also be observed. This suggests that this region contains both low-angle and high-angle misorientations, and the sources of misorientation are relatively complex. By contrast, in the prior-β layer (Figure 9b), the misorientation angle distribution is mainly concentrated near 60°, with a weak secondary peak around 90°. This more discrete distribution is consistent with the multi-variant structure formed during β → α transformation following the Burgers orientation relationship. It suggests that the grain-boundary features in the prior-β layer are mainly controlled by the crystallographic relationship between transformed laths.

**Figure 10:**
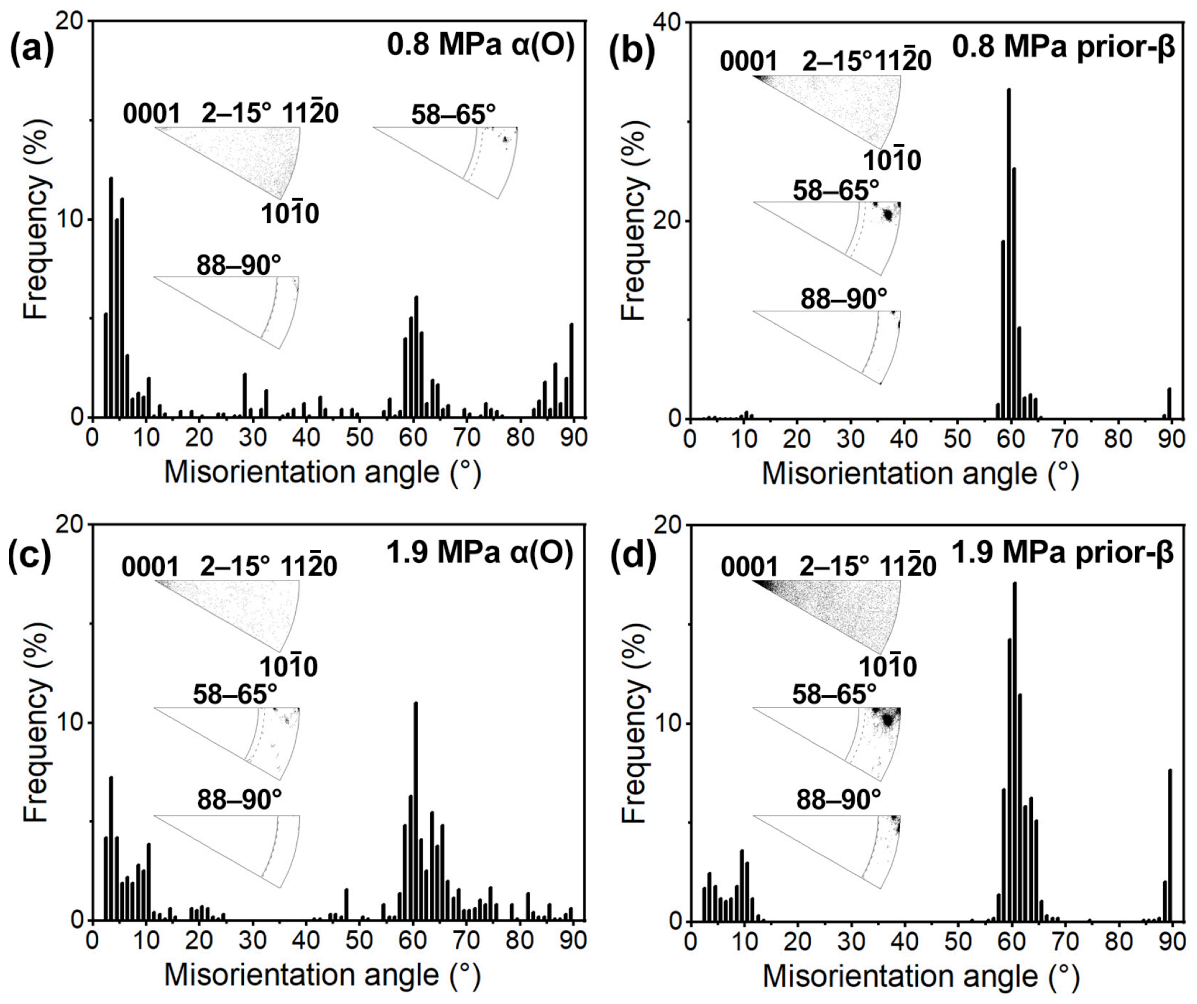
Misorientation angle distributions in different regions of different specimens: (**a**) α(O) layer in the 0.8 MPa specimen; (**b**) prior-β layer in the 0.8 MPa specimen; (**c**) α(O) layer in the 1.9 MPa specimen; and (**d**) prior-β layer in the 1.9 MPa specimen.

A further comparison shows that the prior-β layer in the 1.9 MPa specimen (Figure 10d) retains the main peak near 60°, while the low-angle misorientation distribution becomes more obvious. Together with the stronger lath interweaving shown in Figure 9, this suggests that higher internal pressure leads to a higher degree of retained local orientation gradients and substructures in the prior-β layer. By contrast, the misorientation distribution in the 0.8 MPa specimen (Figure 10b) is more concentrated around the characteristic high-angle peak. This indicates that the misorientation in its prior-β layer mainly comes from the crystallographic relationship between transformed laths, while the fraction of low-angle misorientations is relatively low.

## 4. Discussion

Under simulated LOCA biaxial creep conditions at 900 °C, the N36 zirconium alloy cladding tubes formed a typical layered microstructure of “oxide film–α(O) layer–prior-β transformed layer”. This result indicates that, at this temperature, microstructural evolution is first controlled by the phase-stability partitioning along the thickness direction caused by oxygen diffusion [9,13,14]. The comparison between different internal pressures further shows that internal pressure does not change the basic formation path of this layered structure. Instead, it affects the layered microstructural features indirectly by changing the high-temperature exposure history and the local stress state.

Oxygen diffusion plays a dominant role in the formation of the layered structure. The thickness of the α(O) layer and the oxygen gradient are clearly different under different internal pressures (Figure 3 and Figure 4). The 0.8 MPa specimen shows a thicker α(O) layer and a wider oxygen diffusion-affected region, whereas the 1.9 MPa specimen shows a thinner α(O) layer and a steeper elemental gradient. The macroscopic deformation features further confirm that the 1.9 MPa specimen experienced more severe ballooning deformation compared with the 0.8 MPa specimen, indicating a stronger instability tendency under higher internal pressure. Based on the time–temperature–pressure history during the test, this difference mainly comes from the different high-temperature exposure times. Under higher internal pressure, the specimen becomes unstable earlier, which shortens the effective time window for oxygen diffusion into the metal side [26,27]. Therefore, at 900 °C, the formation and growth of the α(O) layer are mainly controlled by diffusion. The effect of internal pressure is first reflected in its control of the high-temperature history, rather than a direct change in the oxidation mechanism.

In the prior-β transformed layer, typical transformed structures consisting of lath α phase formed under both conditions (Figure 6 and Figure 7), which is consistent with the basic features of β → α transformation [28,29]. However, their morphology and orientation distribution are different. In the 0.8 MPa specimen, the laths are more regularly arranged, showing a more complete colony structure and retaining some prior-β grain outlines. In the 1.9 MPa specimen, the laths are more strongly interwoven, and the orientation distribution is more scattered. Considering its shorter holding time at high temperature, the transformed structure may have been “frozen” at an earlier stage, before sufficient lath interface migration and microstructural regularization could occur. Meanwhile, the stronger local stress state under higher internal pressure may further enhance competition among different variants, leading to a more scattered orientation distribution [18,19].

The KAM maps and misorientation angle statistics further show the differences in local orientation gradients (Figure 8, Figure 9 and Figure 10). The 1.9 MPa specimen has a higher average KAM value and more continuous high-KAM regions, indicating that it retains stronger local distortion and orientation gradients. In contrast, the 0.8 MPa specimen has lower KAM values, and the orientation gradients are more scattered. The misorientation distributions in the prior-β layer show that both specimens have a main peak near 60°, which agrees with the multi-variant transformation features under the Burgers orientation relationship [20,21]. However, under the 1.9 MPa condition, the low-angle misorientation fraction is relatively higher, suggesting greater retention of local substructures and distortion. This difference is consistent with its shorter high-temperature recovery time and stronger stress effect [30].

For the surface oxide film, the TEM results show that the local region is composed of fine equiaxed monoclinic ZrO_2_ grains (Figure 5), with twin-related interfaces [11,31]. This feature reflects local strain accommodation during oxide film growth. However, this conclusion is limited to the observed region and may not be taken as representing the whole oxide film.

From the perspective of accident-tolerant fuel development, suppressing oxygen penetration into zirconium alloys remains an important strategy for improving LOCA tolerance. Previous studies have demonstrated that protective coatings, such as Cr-based coatings and FeCrAl-related materials, can effectively enhance oxidation resistance under high-temperature steam environments. These approaches may delay α(O) layer growth and reduce oxygen embrittlement during LOCA transients. Therefore, combining optimized alloy design with surface protection technologies provides a potential route for improving the accident tolerance of zirconium alloy cladding.

Overall, at 900 °C, the microstructural evolution of the N36 cladding tube is mainly governed by oxygen diffusion and phase transformation kinetics. Oxygen diffusion controls the formation of the layered structure and the thickness of the α(O) layer, while the inner low-oxygen region forms a lath structure through β → α transformation. On this basis, the role of internal pressure is mainly reflected in two aspects. First, it changes the instability behavior and thereby controls the high-temperature exposure time, affecting oxygen diffusion depth and the degree of microstructural regularization. Second, it affects the orientation distribution and distortion retention of the transformed structure through the local stress state. It does not directly change the oxidation or phase transformation process itself.

## 5. Conclusions

(1) Under a steam environment and biaxial loading at 900 °C, a stable layered structure of “oxide film–α(O) layer–prior-β transformed layer” forms in the cross-section of the N36 zirconium alloy cladding tube. This indicates that the microstructural evolution at this temperature is first controlled by the phase-stability gradient produced by oxygen diffusion toward the metal side.
(2) The thickness of the α(O) layer and the oxygen gradient differ clearly under different internal pressures. The 0.8 MPa specimen has a thicker α(O) layer and a wider oxygen-affected region, while the 1.9 MPa specimen shows a thinner α(O) layer and a steeper composition gradient. This difference mainly comes from the different high-temperature exposure times. Higher internal pressure leads to earlier instability, thereby shortening the effective time window for oxygen diffusion.
(3) The prior-β transformed layer is composed of lath α phase under both conditions, but its spatial arrangement and orientation distribution are strongly affected by internal pressure. Under lower internal pressure, the lath structure is more regular and shows a more complete colony structure. Under higher internal pressure, lath interweaving is stronger, and the orientation distribution becomes more scattered.
(4) The KAM maps and misorientation angle statistics show that the prior-β layer in the 1.9 MPa specimen has a higher local orientation gradient and a more obvious low-angle misorientation distribution. In contrast, the 0.8 MPa specimen shows a lower distortion level and more concentrated characteristic high-angle variant boundaries. This indicates that the degree of retained microstructural distortion is higher under higher internal pressure.

## Figures and Tables

**Table 1:** Composition of the N36 cladding tube.

Element	Sn	Nb	Fe	Zr
Content (wt.%)	1.0	1.0	0.3	Bal.

## Data Availability

The original contributions presented in this study are included in the article. Further inquiries can be directed to the corresponding authors.
